# Association between idiopathic hearing loss and mitochondrial DNA mutations: A study on 169 hearing-impaired subjects

**DOI:** 10.3892/ijmm.2013.1470

**Published:** 2013-08-16

**Authors:** VALERIA GUARAN, LAURA ASTOLFI, ALESSANDRO CASTIGLIONE, EDI SIMONI, ELENA OLIVETTO, MARCO GALASSO, PATRIZIA TREVISI, MICOL BUSI, STEFANO VOLINIA, ALESSANDRO MARTINI

**Affiliations:** 1 Bioacoustics Research Laboratory, Department of Neurosciences, University of Padua, I-35129 Padua, Italy; 2 Department of Audiology, University of Ferrara, I-44121 Ferrara, Italy; 3 Department of Morphology and Embriology, University of Ferrara, I-44121 Ferrara, Italy; 4 ENT Surgery, Department of Neurosciences, University of Padua, I-35129 Padua, Italy

**Keywords:** mitochondrial DNA, non-syndromic hearing loss, T961G, mitochondrial DNA variants, polymorphisms

## Abstract

Mutations in mitochondrial DNA (mtDNA) have been shown to be an important cause of sensorineural hearing loss (SNHL). In this study, we performed a clinical and genetic analysis of 169 hearing-impaired patients and some of their relatives suffering from idiopathic SNHL, both familial and sporadic. The analysis of four fragments of their mtDNA identified several polymorphisms, the well known pathogenic mutation, A1555G, and some novel mutations in different genes, implying changes in the aminoacidic sequence. A novel sporadic mutation in 12S rRNA (*MT-RNR1*), not previously reported in the literature, was found in a case of possible aminoglycoside-induced progressive deafness.

## Introduction

Hearing loss (HL) affects 1–3 of every 1,000 newborns; thus, it is one of the most common sensory disorders in humans (1). This condition is caused by environmental factors, such as noise or treatment with ototoxic drugs (e.g., aminoglycoside antibiotics) or genomic alterations. Hereditary HL occurs in the presence of defects either in the nuclear genome, as the 35delG mutation in the gene encoding connexin 26 (*GJB2*), or in mitochondrial DNA (mtDNA). Mutations in mtDNA have been shown to be responsible for both maternally inherited syndromic and non-syndromic HL (NSHL) and play a role in the predisposition to aminoglycoside-induced ototoxicity. Jacobs *et al*(2) demonstrated that in Italy, at least 5% of cases of post-lingual, non-syndromic hearing impairment may be attributed to mtDNA mutations. Furthermore, it has been estimated that up to 67% of patients with mtDNA disorders also manifest sensorineural HL (SNHL) (3). This may be explained by the fact that cells of the cochlea have high oxidative phosphorylation demands, and are thus affected to a greater extent than other cells by a mitochondrial decrease in the protein synthesis rate provoked by mutations in mtDNA.

Non-syndromic SNHL associated with mtDNA mutations is generally progressive (4,5), involving mainly higher frequencies (6–8) and is generally symmetric HL. The onset of HL usually occurs in childhood, is predominantly post-lingual and may be accompanied with vertigo (9) and tinnitus (10,11). There is a high variability in severity ranging from normal hearing to profound deafness, even within families presenting similar genotypes (12–14); this may be due to the fact that the phenotypic effects are a result of several factors and can develop gradually. Some mtDNA variants, in particular in the *MT*-*RNR1* and *tRNA^Ser(UCN)^* genes, have been identified in several cases as the main cause of SNHL, suggesting that these two loci in particular are hotspots for deafness-associated mutations.

The most commonly reported mutations known to cause HL are A1555G (15), 961delT (16–18), C1494T (19), A7445G (20,21), 7472insC (22,23) and A3243G (24,25). These variants together with the use of aminoglycosides or in association with other mutations, either mitochondrial or nuclear, can aggravate the condition of hearing impairment.

In particular, it has been documented that, even though the presence of the mutation, A1555G, itself may induce HL (15), this effect may be worsened in combination with aminoglycoside therapy, as this variant produces a modification in 12 rRNA, making its secondary structure more similar to the corresponding region of *E. coli* 16S rRNA, thus much more vulnerable to the effects of this class of antibiotics (16).

mtDNA variants, as mutations, deletions or insertions, at position 961 in the same *MT*-*RNR1* gene, have been found in patients with SNHL either with or without a history of aminoglycoside therapy (26,27). The T>G substitution in position 961 in particular, has been observed more frequently in hearing-impaired patients compared with controls; thus, it has been suggested to correlate with SNHL (28).

Taking into consideration that thus far, several mutations have been examined and many are yet to be discovered, in our study, we aimed to identify novel potentially pathogenic mtDNA variants and establish the frequency of the known mutations in our cohort of deaf patients.

## Patients and methods

### Patients

In collaboration with the Audiology Clinic at the Hospital of Ferrara, Ferrara, Italy we retrieved data on 169 patients suffering from hearing impairment without known aetiology and some of their close relatives. The present study was composed of 102 females and 67 males, with an average age of 20 years (ranging from 0 to 67 years). Their only clinical feature was HL and they did not present any syndromic sign or other clinical abnormalities, including muscular diseases, diabetes, visual dysfunction or neurological disorders. The analysis referred to the audiological tests data. In the audiometric tests, the severity of hearing impairment was defined by pure-tone threshold average (PTA) in frequencies: 500, 1,000, 2,000 and 4,000 Hz. HL of <20 dB was considered as normal hearing, 21–40 dB mild HL, 41–70 dB moderate HL, 71–90 dB severe HL and >90 dB profound HL. Written informed consent was provided from all study participants prior to enrollment. Any research involving human subjects was conducted in accordance with the ethical standards of all applicable national and institutional committees and with the World Medical Association’s Helsinki Declaration.

### Sequence analysis of mtDNA, secondary structure analysis and sequence conservation

Total DNA was extracted from peripheral blood using the Wizard Genomic DNA Purification kit from Promega (Madison, WI, USA). The analysis and search for the mutations in the genes coding for connexin 26 (*GJB2)*, connexin 30 (*GJB6*) and pendrin (*SLC26A4*) were carried out by the Department of Medical Genetics at the Hospital of Ferrara.

From each subject, four regions corresponding to the mitochondrial genomes coding for 12S RNA (*MT*-*RNR1*), tRNA serine 1 (UCN) (*MT-TS1*), tRNA valine (*MT-TV*), tRNA leucine 1 (*MT-TL1*), tRNA aspartic acid (*MT-TD*) and part of 16S rRNA (*MT-RNR2*), NADH dehydrogenase subunit I (*MT-ND1*), cytochrome *c* oxidase subunit I (*MT-CO1*), cytochrome *c* oxidase subunit II (*MT-CO2*) were PCR-amplified. The PCR products were analysed by direct sequencing in the ABI 3730XL or ABI 3100 sequencing machines at BMR Genomics (Padova, Italy). The sequence data were compared to the revised Cambridge Sequence (rCRS), GenBank accession no. NC_012920 (http://www.ncbi.nlm.nih.gov/nuccore/NC_012920).

The presence and the nature of all identified nucleotide changes (polymorphisms, putative pathogenic variants, mutations) were confirmed through mitomap (http://mitomap.org/MITOMAP) and the Human Mitochondrial Genome Database (http://www.genpat.uu.se/mtDB/) which report published and unpublished data on human mtDNA variations and contain a comprehensive database of the complete human mitochondrial genomes, including sequences from GenBank (16,411 sequences with size >14 kbp) and other sources.

In the subjects harbouring the mutations, A1555G, A3213G, C7792T and T961G, homo/heteroplasmy was determined by electrophoresis on a 1.2% agarose gel following enzymatic digestion as previously described (28).

The RNAfold software (http://rna.tbi.univie.ac.at/cgi-bin/RNAfold.cgi) was used to predict the RNA secondary structure based on minimum energy requirements and base pair probability. The folding of sequences containing novel mutations was compared to the wild-type prediction.

The rCRS and the mitochondrial sequence of 18 different mammals [*Gorilla gorilla, Cavia porcellus, Capra hircus, Bos Taurus*, *Macaca (fascicularis, sylvanus, mulatta, thibetana), Canis lupus familiaris, Felis catus, Equus asinus, Sus scrofa, Mus musculus, Rattus norvegicus, Pongo abelii, Pongo pygmaeus, Pan paniscus, Pan troglodytes*] were aligned using the ClustalW2 sequence alignment program (http://www.ebi.ac.uk/Tools/msa/clustalw2/) to analyse the conservation of the positions of the new sequence variants identified in our patients. We considered the variants conserved with a conservation rate >50%.

## Results

The 169 subjects presented with idiopathic SNHL and no other symptoms. We performed a mutation analysis of four mtDNA fragments corresponding to the hot spots for HL. We detected mutations in *GJB2* in 43 patients and excluded 18 of them from our analysis as they did not show any association with mtDNA variants.

Comparing the mitochondrial genomes to the rCRS, in our cohort of patients, we found 81 different sequence alterations (Table I), including HL-associated A1555G, putatively pathogenic T961G and five other mutations that have never been reported to date. Among the five novel mutations, we hypothesised that one in particular (G786A) may play a role in the onset of aminoglycoside-induced HL.

### A1555G

Three genetically unrelated subjects harboured the homoplasmic A1555G mutation in the *MT*-*RNR1* gene, a mtDNA variant that has been associated with deafness. The subjects were two females and one male with an average age of 47 years suffering from SNHL. The enzymatic digestion of the fragment showed homoplasmy in all cases. The phenotypes were different as one was congenitally deaf, and the other two had the onset of the symptoms at 5 and at 19 years, respectively; unfortunately, none of them could recall any previous exposure to aminoglycosides (Table II).

**Figure 1:**
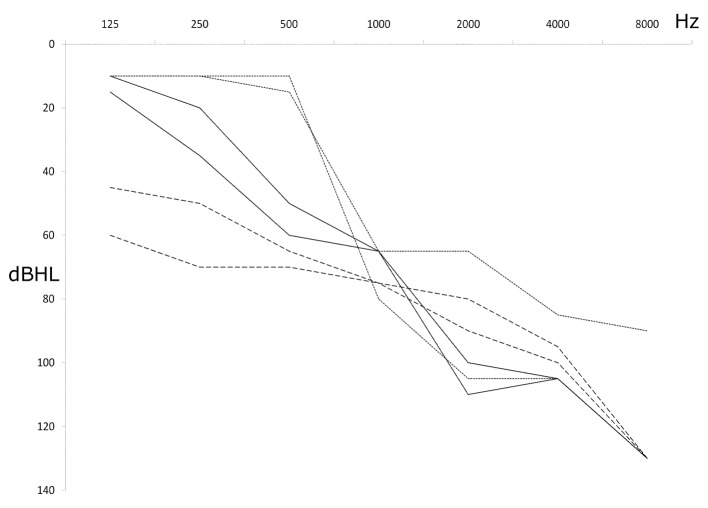
Superimposed audiograms of the three patients with A1555G mutation showing a downsloping trend corresponding to high frequency hearing loss. Hearing measured in decibels Hearing Level (dBHL), frequency in hertz (Hz).

Audiometric examination in all the affected individuals showed a downsloping curve confirming the typical pattern of mitochondrial SNHL, which implicates the loss of high hearing frequencies (Fig. 1). One of these patients with severe progressive hearing impairment harboured two additional mutations whose pathogenicity has yet to be defined: T3504C, a rare variant in the gene coding for *MT-ND1*; and C7471T, a very rare mtDNA variant located in the extraloop of *tRNA^Ser(UCN)^*.

### T961G

Six patients harboured the mutation, T961G, in *MT*-*RNR1*. The phenotypes, as well as the audiometric tests in our T961G cases, were quite disparate as we found two young sisters (mit26 and mit29) with a moderate hearing impairment, whose father and mother were normoacusic even though the latter had the same mtDNA variant. Mit51 showed post-lingual asymmetric progressive HL and in addition to T961G, harboured an additional mutation close to it (C959T) with a low frequency in the databases. Mit116 presented with profound familial congenital SNHL. As for the last two patients, mit178 had hypoplasia of the cochlea and mit186 presented with progressive bilateral HL, which was later diagnosed as partial trisomy of chromosome 6p.

**Figure 2:**
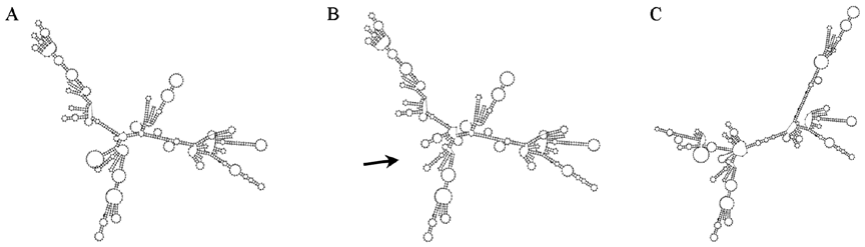
Differences in the predicted *MT*-*RNR1* RNA secondary structure: (A) wild-type, (B) with the T961G mutation, (C) with the novel G786A mutation.

We defined the homoplasmy in all of the cases, with the exception of mit51 whose state could not be determined as the presence of the other mutation in position 959 prevented the *Aci*I restriction enzyme digestion. The comparison of the RNA secondary structure determined by this mutation shows a clear difference with the wild-type one (Fig. 2).

### Novel mutations

We detected novel sequence variants not present in the literature or in mitochondrial databases (Table III), including C712A and the heteroplasmic G786A in *MT*-*RNR1*, A3213G in *MT-RNR2*, C7534T in the D-loop of *TRND* (*tRNAAsp*) and in the *MT-CO2* gene, A7746G, which produces an aminoacidic change in translation. All the mutations were recorded in mitomap (http://www.mitomap.org/bin/view.pl/MITOMAP/VariantSubmissionList) and numbered from 20111230001 onwards. In a phylogenetic analysis, we compared the human nucleotide variants with other 18 different mammals and found a conservation rate of >50% for variants 712, 786 and 3213.

Among the novel mutations, we particularly considered the heteroplasmic G786A in mit7, a 39-year-old female. Her parents and sister were normoacusic and did not harbour any mutations either in mtDNA or in HL-associated genes. She suffered from asymmetric progressive SNHL and had been treated with streptomycin in her childhood. In our alignment analysis, position 786 in the *MT*-*RNR1* gene was quite conserved (14/18); moreover, the mutated secondary structure prediction showed to be different compared to the wild-type one (Fig. 2).

In *MT*-*RNR1* we also found the mutation, C712A, which may have an effect on HL as the site shows a 100% conservation even if no differences in the RNA structure of the gene are detectable. The patient harbouring this variant, a 13-year-old subject with mild SNHL (mit184), harboured two additional mutations of A1811G: a polymorphism and a quite rare C>A mutation in the evolutionarily conserved position 3546 in the *MT*-*ND1* gene.

A3213G in *MT-RNR2* was detected in a young girl from Morocco harbouring several other variants (A3348G, G3591A, A3714G, G7642A and G7805A) with congenital profound SNHL. This was conserved and had a different RNA structure.

Mit145 harboured C7534T in the D-loop of *tRNAAsp* together with G709A and the rare A8014T mutation in *MT-CO2*. The other novel variant, A7746G, detected in the *MT-CO2* gene, not conserved, was found in a 5-year-old boy also harbouring the T980C variant in *MT*-*RNR1*. A7746G presents a missense mutation with the aminoacidic change Asn>Ser in the subunit of cytochrome *c* oxidase (complex IV).

### Low frequency mutations

We identified several other variants that may be associated with hearing impairment, presenting a low frequency in mtDB and mitomap (Table IV). Among these, we preferably considered the mutations in subjects presenting audiograms compatible to a mitochondrial mutation HL diagnosis, in conserved positions and with a frequency <0.05%, such as: i) the mutation A>G in the conserved position 644 (0,04% in mtDB) in *MT-TF*, located in the acceptor stem of *tRNAPhe*. 644A>G found in a 13-year-old girl with SNHL, harbouring the polymorphisms, G709A, G1888A and C7873T; ii) T721C in *MT-RNR1*. This 36-year-old female had progressive HL which began at age 22; the RNA structure though did not seem to differ from the wild-type one; in fact, we eventually found the same mutation in a 34-year-old male heterozygotic for connexin 26 35delG who was normoacusic; iii) T1119C in *MT*-*RNR1* found in mit110: a 36-year-old patient with progressive post-lingual bilateral SNHL which began at age 33; the RNA showed a different structure; iv) C3342T in the *ND1* gene in two deaf sisters harbouring both the additional mutation T7961C; v) A3808G a mutation in a conserved site found in two sisters with audiograms compatible to mitochondrial deafness; vi) A3847G in a case of a 37-year-old female whose mild sporadic hearing impairment began in her thirties; vii) A7720G in *MT-CO2* in a 3-year-old subject presenting with mild progressive hearing impairment; viii) C7792T in *MT-CO2*, observed in a 42-year-old male with progressive hearing impairment which began in his twenties, presenting with moderate to severe symmetric impairment confirmed by a downward overlapping audiogram; ix) G7830A G7984A together with G709A and G1888A in a 45-year-old female with moderate HL at high frequencies.

### Connexin 26 and mtDNA mutations

We searched for a correlation between mutations in connexin 26 and mtDNA mutations. Eighteen patients only harboured mutations in the *GJB2* gene and 25 of them harboured both the *GJB2* and mtDNA variants (10 of whom were homozygotic for 35delG).

In our subjects, we noticed a higher presence of the G3915A polymorphism, as 5 out of the total 7 probands with this polymorphism in *NDI* were associated with *GJB2* mutations. In two siblings with SNHL and 35delG/35delG in *GJB2,* we identified the missense mutation, T3308C (Met>Thr), at the highly conserved amino acid position 1 in *MT-ND1*. Among the patients with homozygotic 35delG in *GJB2* we found some rare mutations that may worsen their condition of hearing impairment (Table V). The mutations found were A3447G, C3903T, A7717G and G8027A, all in conserved positions in the genes *MT-ND1* and *MT-CO2*. In the literature these were found to be more involved in Leber’s hereditary optic neuropathy (LHON) than in HL. Another patient homozygotic for 35delG showed two additional variants: the missense mutation, A3505G, causing the Thr>Ala substitution in *MT-ND1* and the conserved T1243C mutation in the *MT*-*RNR1* gene.

## Discussion

In the present study, we analysed four fragments of mtDNA in 169 subjects with non-syndromic SNHL, both familial and sporadic without a clear aetiology. We compared our data with the DNA of some of their relatives who were normoacusic in order to define whether the mutations were sporadic or genetically transmitted. We also considered the mutations in the *GJB2*, *GJB6* and *SLC26A4* genes which are recognised to be among the most frequent causes of hearing impairment. In total, 43 patients harboured *GJB2* mutations and 18 were affected by *GJB2* mutations only (no mtDNA mutations). We thus decided to exclude this group from our analysis.

The hearing-impaired patients showed a wide range of penetrance, severity and age-at-onset of HL. We searched for mutations in the regions corresponding to the hotspots for deafness: the *MT*-*RNR1* and the *MT-TS1* genes, as the presence of mutations in these two genes in particular, is known to cause both syndromic and non-syndromic forms of hearing impairment; we also focused on the region of *MT*-*TL1* as previous studies report its possible role in non-syndromic disease (29). In order to establish the potential pathogenicity of the mutations encountered, we analysed the evolutionary conservation comparing our sequences to those of other organisms. Furthermore, considering that the biological functions of 16S rRNA and tRNAs and other structural RNAs are dictated by their three dimensional structures, we analysed the possible RNA secondary structure of the mutated samples and predicted the folding using the Vienna RNA package. Our aim was to detect and correlate the frequency of mtDNA alterations in the cases of deafness showing the typical audiological manifestations of mitochondrial SNHL.

In our cohort of patients, we identified three subjects harbouring the A1555G mutation. This mutation in the *MT*-*RNR1* gene is one of the most common mtDNA variants associated with both non-syndromic progressive SNHL and aminoglycoside-induced SNHL. Sequence analysis of the *MT*-*RNR1* gene in our subjects identified three genetically unrelated individuals harbouring the A1555G mutation who showed the typical mitochondrial HL audiometric features. The incidence of the mutation in hypoacusic subjects was 2%, a little lower than the one recognised by Berrettini *et al*(29) in 2008, but similar to the data presented in the studies by Jacobs *et al*(2) and Lingala *et al*(30). We could not state if the use of aminoglycosides had any effect on these subjects as they could not recall any exposure to antibiotics in the past; however, one of these patients with severe and progressive HL harboured a novel mutation in position 7471 in *tRNA^Ser(UCN)^*, close to position 7472, which has shown to cause both syndromic and non-syndromic deafness (31), suggesting that this variant somehow functions as a modifier, in synergy with the primary mutation, thus modulating its phenotypic manifestations as observed for other tRNA mutations (32).

We identified another mutation in the *MT*-*RNR1* gene: seven patients harboured the T961G mutation with a frequency corresponding to data reported in the literature. Its pathogenicity is quite controversial: the mutations at position 961 have been detected in subjects affected by aminoglycoside-induced NSHL. The delT961Cn mutation is more frequent in Caucasian and Asian subjects (16,26,27,33), as well as the 961C insertion (17,19,27,28), T961C mutation in Chinese subjects (4) and T961G mutation in the Caucasian population (17). In a previous study, Li *et al*(28) found the T961G substitution in 5/164 hearing-impaired paediatric patients of Caucasian descent without a history of exposure to aminoglycoside, while the 226 Caucasian and 324 Chinese control subjects did not harbour this mutation; thus, it was hypothesised that this variant may be associated with SNHL. In contrast to these results indicating a possible pathogenic nature of the mutations around position 961 in NSHL and aminoglycoside-induced HL, Herrnstadt *et al* stated that it could be a typical polymorphism of the H2 haplogroup (34). The localization of position 961 is at the C-cluster of the region between loop 21 and 22 of *MT*-*RNR1*(35); compared with A1555G this region is not evolutionarily conserved and is in fact highly polymorphic in mammalian interspecies comparisons. Its function is not well defined; in particular, its pathogenic mechanisms of action in the predisposition of carriers to aminoglycoside toxicity remain unclear (17,36). Elstner *et al* performed a single nucleotide polymorphism (SNP) analysis of the nucleotide 961 in a control group of 320 German samples, finding six T>C and five T>G nucleotide changes (37). Thus, the effects of this mutation have yet not been defined; we confirmed this mutation in our screening; six out of seven patients with T961G showed variable degrees of hearing impairment, suggesting at least a minor role in the HL onset; however, at the same time the mother of two hearing-impaired children harbouring the same mutation did not present with HL. Thus, it can be hypothesised that T961G is either a polymorphism, or a pathogenic mutation with an extremely low penetrance.

One of the subjects in our cohort of patients harboured a novel mutation in position 786 in *MT*-*RNR1*. She did not harbour any other mutations in the genes usually associated with HL or any malformations. This alteration, in our opinion, could be the reason of her HL since its conserved site in the hotspot gene for HL and also as the RNA structure shows a clearly different folding compared with the wild-type one, suggesting a possible malfunctioning of the ribosome. From a clinical point of view, the patient presented with sporadic progressive SNHL with post-lingual onset; her audiometry was compatible with mitochondrial-associated HL and the fact that she was treated with aminoglycosides in the past confirms our hypothesis.

In our patient cohort, some other novel mtDNA variants in genes that are not usually involved in HL or have an association with other pathological conditions were recorded, though their exact role is unclear; thus, they should be investigated, further studied and compared with new cases.

We suggest that some of the rare mutations harboured by patients with audiometric data compatible with a mitochondrial HL are possible candidates for genetic risk factors of NSHL. Among these, we considered T1119C in *MT*-*RNR1*. We suggest that this variant detected in 36-year-old female may be responsible for her mild progressive bilateral SNHL, which began three years earlier. T1119C, already found in four subjects suffering from hearing impairment by Li *et al*(4), located in a conserved site and presenting with a different RNA structure, may be the cause of HL at high frequencies, confirmed by an audiogram. It should be noted that the late onset and gradual worsening of the impairment may reflect the tendency of the mitochondrion to accumulate mutations with aging due to its genomic instability.

Of note, we observed the non-pathogenicity of the T721C mutation in *MT*-*RNR1* that we thought could be responsible for the progressive HL of a 36-year-old female which began at age 22. In fact, we eventually detected the same mutation in a 34-year-old male heterozygotic for connexin 26 35delG who was normoacusic.

In patients harbouring mutations in the most common HL-associated genes (connexin 26), we focused on the mtDNA mutations, in particular T3308C, which results in a change in the initiation codon of NADH dehydrogenase. In a study on mutant cells, Li *et al*(38). demonstrated that T3308C induces a significant decrease in the levels of *MT-ND1,* resulting in a decreased complex I activity; furthermore, the T3308C mutation may also alter the hydrophobicity and antigenicity of the N-terminal peptide of *MT-ND1*(39). These facts suggest that a combination of a mtDNA mutation with other genomic DNA mutations may increase the penetrance of deafness.

In conclusion, our data confirm a frequency of 2% for the A1555G mutation and its role in NSHL; however, the pathogenicity of all the other mtDNA variants encountered should be established: the variability of the frequency in different haplogroups, the occurrence in normal hearing individuals and the correlation with other conditions and mutations should be taken into account; thus, further genetic and functional studies are required in order to define their possible additional correlation with NSHL and/or aminoglycoside-induced HL.

## Figures and Tables

**Table I:** mtDNA alterations detected and conservation.

	Hypoacusic (n=168)	% hypoacusia with no *GJB2* mutation *(*n*=*150)	Cons.	mtDB % on 2704	Mitomap % on GenBank 16411
Polymorphism					
G709A	18	12	Yes	16.4	13.79
T710C	1	0.67	No	0.89	1.16
A750G	166	100	Yes	99.18	97.64
G930A	1	0.67	No	2.25	2.42
G951A	3	2	No	0.29	0.61
T1189C	5	3.33	Yes	3.85	4.42
T1243C	6	4	Yes	2.11	1.44
A1438G	163	100	Yes	96.89	94.99
T1700C	4	2.67	No	0.18	0.74
G1719A	15	10	No	4.10	4.41
A1811G	12	8	No	7.54	8.49
G1888A	9	6	No	5.32	6.19
T3336C	1	0.67	Yes	0.33	0.64
A3348G	1	0.67	Yes	1.74	0.80
T3394C	2	1.33	Yes	1.44	1.64
T3396C	1	0.67	No	0.22	0.83
A3447G	1	0.67	Yes	0.44	0.52
A3480G	4	2.67	Yes	4.85	5.26
A3505G	3	2	No	2.07	1.25
G3591A	2	1.33	No	0.74	0.53
T3644C	1	0.67	Yes	0.48	0.67
G3666A	1	0.67	No	2.15	2.14
G3705A	1	0.67	No	1.15	1.21
A3720G	1	0.67	Yes	0.70	0.65
T3847C	1	0.67	No	0.26	0.74
G3915A	7	4.67	Yes	0.81	1.41
G7337A	2	1.33	No	0.55	0.97
G7521A	3	2	No	5.62	5.45
A7768G	4	2.67	Yes	2.22	2.16
G7805A	1	0.67	No	1.37	0.86
G7853A	1	0.67	No	1.66	1.15
T7961C	2	1.33	No	0.18	0.72
G8027A	1	0.67	No	2.14	3.22
Possible HL-associated mutations					
T961G	6	4	No	0.18	0.37
HL-associated mutations					
A1555G	3	2	Yes	0.44	nd
Novel mutations					
C712A	1	0.67	Yes	nd	nd
G786A	1	0.67	Yes	nd	nd
A3213G	1	0.67	Yes	nd	nd
C7534T	1	0.67	No	nd	nd
A7746G	1	0.67	No	nd	nd
Rare mutations					
A644G	1	0.67	Yes	0.04	0.07
T721C	2	1.33	No	0.18	0.24
T742C	1	0.67	No	0.07	0.06
A813G	1	0.67	No	1.63	0.49
C867T	1	0.67	No	0.04	0.03
A942G	2	1.33	No	0.11	0.09
C959T	1	0.67	No	nd	0.13
T980C	2	1.33	No	0.51	0.46
A1118G	1	0.67	Yes	0.04	nd
T1119C	1	0.67	Yes	0.96	0.45
T1193C	1	0.67	Yes	0.29	0.26
C1405T	2	1.33	Yes	0.04	nd
T1406C	2	1.33	Yes	0.37	0.32
A1618G	1	0.67	Yes	0.04	0.03
A1708T	1	0.67	Yes	0.04	0.01
T3308C	2	1.33	Yes	0.81	0.01
C3342T	2	1.33	No	0.04	0.06
C3388A	1	0.67	Yes	0.07	0.07
T3504C	1	0.67	No	nd	0.12
A3511G	1	0.67	No	0.04	0.14
C3546A	1	0.67	Yes	0.11	0.05
T3645C	1	0.67	No	0.18	0.15
A3672G	1	0.67	Yes	0.07	0.14
A3714G	2	1.33	Yes	0.15	0.17
C3741T	1	0.67	No	0.18	0.20
C3792T	1	0.67	No	nd	nd
A3808G	2	1.33	Yes	0.04	0.07
C3903T	1	0.67	Yes	0.04	nd
C3936T	1	0.67	Yes	0.07	0.04
A7385G	1	0.67	Yes	0.63	0.40
T7440G	1	0.67	No	nd	nd
C7471T	1	0.67	No	nd	0.04
G7642A	1	0.67	No	0.30	0.25
T7645C	2	1.33	No	0.22	0.29
T7705C	2	1.33	No	0.18	0.40
A7717G	1	0.67	Yes	nd	nd
A7720G	1	0.67	Yes	nd	0.01
C7792T	1	0.67	Yes	nd	0.04
G7830A	1	0.67	No	0.15	0.10
C7873T	1	0.67	No	0.15	0.12
G7984A	1	0.67	No	0.07	0.07
A8014T	1	0.67	No	0.15	0.32

mtDNA, mitochondrial DNA; HL, hearing loss; nd, not determined; Cons., conserved.

**Table II:** Patients harbouring the hearing loss-associated A1555G mutation.

Patient	Gender	Age (years)	Homo/heteroplasmy	2d	*GJB2*	*GJB6*	*SLC26A4*	Age of onset (years)	PTA dx	PTA sn	Family history of HL	Other mtDNA mutations	Type of line in Fig. 1
Mit76	F	47	Homo	Yes	wt	wt	wt	5	80	81.2	No, sporadic	C7471T; T3504C	Continuous
Mit114	F	44	Homo	Yes	wt	wt	wt	19	56.2	75	No, sporadic	No	Dotted
Mit140	M	50	Homo	Yes	wt	wt	wt	At birth	80	82.5	nd	No	Dashed

2d, differences in the secondary structure; PTA, pure-tone threshold average; dx, right; sn, left; HL, hearing loss; mtDNA, mitochondrial DNA; F, female; M, male; wt, wild-type; nd, not determined.

**Table III:** Novel mutations.

Mutation	Homo/heteroplasmy	Gene	*GJB2*	*GJB6*	*SLC26A4*	Age of onset (years)	PTA dx	PTA sn	Family history of HL	Other mtDNA mutations	Conserved	Patient	Age (years)	Gender	Notes	Origin	2d
C712A	Homo	*RNR1*	wt/wt	wt/wt	nd	0	35	40	Uncle?	A1811G	Yes	Mit184	13	M	SNHL Perinatal asphyxia? Normoacusic brother	Italy Sardinia	Yes
G786A	Hetero	*RNR1*	wt/wt	wt/wt	nd	Post-lingual	85	72.5	No, sporadic	No	Yes	Mit7	39	F	SNHL progressive	Italy	Yes
A3213G	nd	*RNR3*	wt/wt	wt/wt	6,7, 8, 10,19 wt	Congenital	95	95	No, sporadic	A3348G; G3591A; A3714G; G7642A; G7805A	Yes	Mit70	4	F	SNHL	Morocco	No
A7746G Asn→Ser	Homo	*CO2*	wt/wt	wt/wt	wt/wt	0	61	59	No, sporadic	T980C	No	Mit100	4	M	SNHL otitis CT ok	Italy	Yes

PTA, pure-tone threshold average; dx, right; sn, left; HL, hearing loss; mtDNA, mitochondrial DNA; 2d, differences in the secondary structure; wt, wild-type; nd, not determined; F, female; M, male; SNHL, sensorineural HL; CT, computed tomography.

**Table IV:** Rare mutations.

Mutation	Gene	*GJB2*	*GJB6*	*SLC26A4*	Age of onset (years)	PTA dx	PTA sn	Family history of HL	Other mtDNA mutations	Conserved	Patient	Age (years)	Gender	Notes
A644G	*RNR1*	wt/wt	wt/wt	nd	nd	40	43.75	No	G709A; G1888A; C7873T	Yes	Mit22	13	F	Italy, SNHL, microhematuria, close to 642 (T>C = HL)
T721C	*RNR1*	wt/wt	wt/wt	wt/wt	22	89	81	No, sporadic	No	No	Mit109	36	F	Italy, SNHL
T721C	*RNR1*	35delG/wt	wt/wt	nd	No	0	0	No, sporadic	No	No	Mit162	34	M	Italy, normoacusic
A813G	*RNR1*	nd	nd	nd	nd	70	70	No, sporadic	No	No	Mit58	49	M	Italy, SNHL, MRI ok
T1119C	*RNR1*	wt/wt	wt/wt	wt/wt	33	28	25	Maybe mother	G709A; T1243C	Yes	Mit110	36	F	Italy, progressive SNHL
C3342T	*ND1*	wt/wt	wt/wt	wt/wt	nd	61	60	nd	T7961C	No	Mit158–159	45–49	M-F	Italy, SNHL, brothers, medium + high frequencies
T3504C	*ND1*	wt/wt	wt/wt	wt/wt	5	80	81.25	No, sporadic	A1555G; C7471T	No	Mit76	47	F	Italy, SNHL
A3511G Thr→Ala	*ND1*	wt/wt	wt/wt	wt/wt	0	54	79	Adopted	G709A; T1193C T3394C; G3591A	No	Mit87	8	F	India
C3546A	*ND1*	wt/wt	wt/wt	nd	0	35	40	Uncle?	C712A; A1811G	Yes	Mit184	13	M	Sardinia, SNHL perinatal asphyxia. Normoacusic brother
T3644C Val→Ala	*ND1*	wt/wt	wt/wt	nd	1.2	122.5	122.5	No, sporadic	T3336C; T3396C	Yes	Mit61	10	F	Ecuador, SNHL not progressive
T3645C	*ND1*	nd	nd	nd	nd	Moderate	Moderate	Familial	G1719A; T3645C; G7521A	No	Mit151	56	F	Italy, SNHL
A3672G	*ND1*	wt/wt	wt/wt	wt/wt	0	nd	nd	No, sporadic	A1811G; T7705C	Yes	Mit167	0	F	Italy, SNHL
G3705A	*ND1*	wt/wt	wt/wt	wt/wt	2.5	115	16	No, sporadic	G709A; G1888A; G3705A	No	Mit127	nd	M	Albania, SNHL
A3720G	*ND1*	wt/wt	wt/wt	nd	4	71	61	No	A1811G; A3720G	Yes	Mit129	44	F	Italy, SNHL progressive, otitis
C3741T	ND1	wt/wt	wt/wt	wt/wt	6 months	nd	nd	No, sporadic	T980C; A1811G	No	Mit180	3	M	Italy, SNHL CT/MRI ok Normoacusic brother
A3808G	ND1	wt/wt	wt/wt	nd	nd	80	82.5	nd	G1719A	Yes	Mit24–25	41–46	F	Italy, SNHL, sisters
T3847C	ND1	nd	nd	nd	30	38.75	38.75	No, sporadic	No	Yes	Mit72	37	F	Italy, SNHL
C3936T	ND1	wt/wt	wt/wt	wt/wt	2.5	34	75	No, sporadic	No	Yes	Mit153	4	M	Italy, SNHL
A7385G	CO1	wt/wt	wt/wt	nd	nd	70	35	No, sporadic	A7768G	Yes	Mit57	51	M	Italy, SNHL
T7440G Ser→Ala	CO1	wt/wt	wt/wt	nd	7	nd	nd	No, sporadic	G709A; G1888A; T7440G	No	Mit143	8	M	Italy SNHL IQ ok. premature
C7471T	S^(UCN)^	wt/wt	wt/wt	wt/wt	nd	80	81.2	No, sporadic	A1555G; T3504C	No	Mit76	47	F	Italy SNHL, close to 7,472 known to cause HL
G7642A	CO2	wt/wt	wt/wt	6, 7, 8, 10, 19 wt	Congenital	95	95	nd	A3213G; A3348G; G3591A; A3714G; G7805A	No	Mit70	4	F	Morocco, SNHL negative anamnesis
A7720G	CO2	wt/wt	wt/wt	wt/wt	nd	19	26	No, sporadic	No	Yes	Mit89	3	M	Not Italy, SNHL
C7792T	CO2	wt/wt	wt/wt	nd	22	69	68	nd	G8020A	Yes	Mit142	42	M	Italy SNHL progressive
G7805A Val→Ile	CO2	wt/wt	wt/wt	6, 7, 8, 10, 19 wt	Congenital	95	95	nd	A3213G; A3348G; G3591A; A3714G; G7642A	No	Mit70	4	F	Morocco, SNHL negative anamnesis
G7830A Arg→His	CO2	wt/wt	wt/wt	wt/wt	nd	60	60	nd	G709A; G1888A; G7984A	Yes	Mit94	46	F	Italy, SNHL
G7853A	CO2	nd	nd	nd	nd	19	26.25	nd	G709A; G1888A; G7853A	No	Mit187	5	M	Italy, SNHL
C7873T	CO2	wt/wt	wt/wt	nd	nd	40.00	43.75	No	A644G; G709A; G1888A; C7873T	No	Mit22	13	F	Italy, SNHL
G7984A	CO2	wt/wt	wt/wt	wt/wt	nd	60	60	nd	G709A; G1888A; G7830A	No	Mit94	46	F	Italy, SNHL

PTA, pure-tone threshold average; dx, right; sn, left; HL, hearing loss; mtDNA, mitochondrial DNA; wt, wild-type; nd, not determined; SNHL, sensorineural HL; CT, computed tomography.

**Table V:** mtDNA and *GJB2* mutations.

Sample	mtDNA mutation	Gene	*GJB2*	Notes
Mit1–2	C1405T	RNR1	35delG/35delG	Severe SNHL, homozygous twins
Mit46–47	T3308C	ND1	35delG/35delG	Mild-moderate SNHL, brothers
Mit73	A3447G; G8027A	ND1; CO2	35delG/35delG	Profound SNHL, familial
Mit74	G3915A	ND1	35delG/35delG	SNHL
Mit135	T7645C	CO2	35delG/35delG	Profound SNHL
Mit154	T1243C; A3505G; C3792T	RNR1; ND1	35delG/35delG	Profound SNHL
Mit185	A942G; T3394C	RNR1; ND1	35delG/35delG	Moderate SNHL, progressive
Mitpds7	C3903T; A7717G	ND1; CO2	35delG/35delG	SNHL, congenital, familial
Mit4	G3915A	ND1	L90P/M34T	SNHL, congenital
Mit5	G3915A	ND1	35delG/L90P	SNHL, congenital
Mit6	G3915A	ND1	35delG/L90P	SNHL, congenital
Mit32	G1719A	RNR2	35delG/wt	SNHL, progressive, familial
Mit49	T1189C; A1811G; A3480G	RNR1; RNR2; ND1	R127H/wt	Profound SNHL, familial, onset at age 4
Mit83–115	G7521A	TD	L90P/wt	EVA, transmissive HL, onset at age 3, brothers
Mit123	G3915A	ND1	M34T/wt	Moderate SNHL
Mit133	A1811G; A3480G	RNR2; ND1	35delG/R184P	Profound SNHL, congenital
Mit139	C959T; G1719A	RNR1; RNR2	wt/35delG	Normoacusic
Mit145	G709A; C7534T; A8014T	RNR1; TD; CO2	wt/del120E	Normoacusic
Mit155	A7768G	CO2	wt/M34T	SNHL, sisters
Mit156	A7768G	CO2	35delG/M34T	SNHL, sisters
Mit162	T721C	RNR1	35delG/wt	Normoacusic

mtDNA, mitochondrial DNA; HL, hearing loss; SNHL, sensorineural HL; wt, wild-type; EVA, enlarged vestibular aqueduct.
